# Breaking Barriers Amid the Pandemic: The Status of Telehealth in Southeast Asia and its Potential as a Mode of Healthcare Delivery in the Philippines

**DOI:** 10.3389/fphar.2021.754011

**Published:** 2021-11-08

**Authors:** Aitana Dy Macariola, Theara Mae Capacion Santarin, Ferianne Joy Manday Villaflor, Leofe Marie Guintos Villaluna, Rea Shane Leonora Yonzon, Jamie Ledesma Fermin, Shaira Limson Kee, Nouar AlDahoul, Hezerul Abdul Karim, Myles Joshua Toledo Tan

**Affiliations:** ^1^ Department of Natural Sciences, University of St. La Salle, Bacolod, Philippines; ^2^ Yo-Vivo Corporation, Bacolod City, Philippines; ^3^ Department of Electronics Engineering, University of St. La Salle, Bacolod, Philippines; ^4^ Faculty of Engineering, Multimedia University, Cyberjaya, Malaysia; ^5^ Department of Chemical Engineering, University of St. La Salle, Bacolod, Philippines

**Keywords:** telehealth, telemedicine, Philippines, southeast asia, COVID-19, pandemic, industry – 4.0, medicine 4.0

## Introduction

Southeast Asia (SEA) is a geographical bloc that is sociologically, politically, and economically diverse. This diversity has led to heterogeneity in levels of development of healthcare systems in its member states (Chongsuvivatwong et al., 2011). Nevertheless, countries in SEA share joint commitments, particularly in achieving Universal Health Coverage. Their efforts toward this goal has progressed significantly, as evidenced by the increasing availability of preventive and curative care services across SEA (Van Minh et al., 2014).

As it is, the healthcare system of SEA struggles to meet the ever-changing demands of its aging population that has become more prosperous and aware of its human rights (Chongsuvivatwong et al., 2011). The problem is the insufficient healthcare workforce of SEA, with only 1.93 doctors per 10,000 population in Cambodia as the lowest, and 22.94 doctors per 10,000 population in Singapore as the highest, according to the World Health Organization (WHO) (Geneva: World Health Organizations, 2021). In the report, eight out of 11 SEA countries (Cambodia, Indonesia, Laos, Myanmar, Philippines, Thailand, Timor-Leste, and Vietnam) have doctor-population ratios lower than the WHO recommendation of 10 doctors per 10,000 population. This translates to immense amounts of work for doctors serving patients in the region.

Excessive workload leads to physician burnout, impairing their efficiency and increasing medical errors (Michtalik et al., 2013). Moreover, healthcare facilities, particularly public hospitals, are frequently overcrowded in low-resourced SEA countries. The situation is due to a shortage of beds, staff, supplies, and diagnostic equipment (Lim et al., 2014), which causes delays in care, and leads to many patients leaving without being examined. Since most individuals in urban areas have hectic schedules, many avoid obtaining medical treatment entirely (Taber et al., 2015). This medical avoidance can lead to delays in diagnosis and treatment, resulting in higher morbidity and mortality in various cases (Moser et al., 2006).

Moreover, health workers in SEA are primarily concentrated in urban areas because of greater prospects and pay in these places, leaving rural areas understaffed (Kanchanachitra et al., 2011; Nair and Webster, 2013). This compels rural folk to seek medical treatment in cities when medical specialists are not accessible in their locality. This problem is often accompanied by distant and lengthy travel, additional expenses, and a lack of reliable transportation (Douthit et al., 2015).

Healthcare-associated infections with a pooled prevalence of 9.0% also pose a challenge in patient care throughout SEA (Ling et al., 2015). When visiting healthcare facilities, an individual is exposed to pathogens from various sources. For instance, if an individual infected with measles is also present in the waiting room, the probability of transmission is more than 10% on 59% of occasions (Beggs et al., 2010). Children, in particular, have an increased risk of direct infections from touching toys and playing closely with other sick children in waiting rooms (Merriman et al., 2002). As a result, some individuals decide not to seek medical attention to avoid being around ill individuals.

With progress in technology over the past decades, access to services have indeed improved, especially in the healthcare and communication industries. Digital health drivers brought by Industry 4.0 have immensely enhanced healthcare services (Ting et al., 2020; Ye, 2020), making them faster and more efficient globally. In fact, in technologically advanced countries, healthcare has started shifting toward remote services, making it easier to receive medical care (Kruse et al., 2017). This, consequently, has given rise to the telehealth industry.

Telehealth is integrated into the system to make healthcare more accessible and affordable. In the United States (US), the number of annual telehealth visits increased from 206 in 2005 to 202,314 in 2017 (Barnett et al., 2018). Furthermore, the outbreak of the COVID-19 pandemic in 2020 also caused an acceleration in telehealth usage. In fact, the increase in telehealth usage coincided with the rise in COVID-19 cases (Weber et al., 2020). Compared to the same month in 2019, US data showed a 154% increase in telehealth usage in March 2020 (Koonin et al., 2020). It seems as though the pandemic acted as a catalyst for the modernization of healthcare.

Telehealth has allowed doctors to provide health services remotely (Dorsey and Topol, 2016). Its advantages are not only concentrated on patient wellbeing but also on that of healthcare workers. It makes use of health information technology (HIT) and remote patient monitoring (RPM) to deliver consultations at a distance (Ye, 2020). With the help of advancements in the internet of things (IoT), biomedical sensor technologies, and modern communication, patient monitoring at home has become possible (Malasinghe et al., 2019; Ahmad et al., 2021). These medical devices, which ease the workloads of healthcare workers, are innovations brought about by biomedical engineers. Biomedical engineering (BME) is a discipline that uses engineering principles in medicine and biology to produce advanced medical technologies that improve healthcare systems (Al Asif et al., 2018). Biomedical engineers are essential members of the health workforce in countries with well-established healthcare systems. In fact, according to a study by Fermin and Tan (2020), healthcare improvements can be observed along with an increase in BME research in ASEAN countries. As seen from the same study, the products of this field, which include telehealth, are fundamental drivers for an improved healthcare system.

The rapid advancements in medical technologies reinforce the already expanding demand for Telehealth globally (Swanepoel et al., 2010). In 2017, the WHO reported that there would be a significant increase in healthcare expenses in Asia in the next 10 years (Raghavan et al., 2021). Projections predict a 1.2% annual growth per capita in health expenditures in Brunei, and 7.4% growth in Myanmar from 2014 to 2040 (Dieleman et al., 2017). This increase is mainly driven by population growth, indicating that healthcare services will also grow in demand. Raghavan et al. (2021) predicted that by 2050, the escalating population in Asia would increase the demand for healthcare facilities. With this significant growth in the population, telehealth could become a viable solution for these problems in the future.

The telehealth industry is bound to become the solution to the aforementioned crises. Most SEA countries have varying telehealth guidelines that primarily focus on ethical and clinical aspects but fail to discuss the technology needed to deliver these services (Sabrina & Defi, 2021). Thus, detailed technology guidelines should be established. These guidelines will enable countries to have a basis for implementing telehealth and to ensure high-quality care. Government agencies should also implement necessary measures to fortify the telehealth industry and fill the overwhelming healthcare gap.

## The Technological Drivers of Telehealth

Telehealth is the result of the entry of advanced technology into the health industry. With the upgrade and fusion of these technologies, the potential of telehealth as an ecosystem within the larger global healthcare sphere is eminent.

First, AI is utilized in electronic health records, health monitoring, and many more. Computers in clinical imaging can assist doctors in the interpretation of medical images. It is commonly used in Radiology and has increased the accuracy, efficiency, and productivity in the specialty (Jalal et al., 2019).

Jagadeeswari et al. (2018) recently reviewed the emerging technologies in a personalized healthcare system, including IoT, cloud computing, big data analytics, and mobile technology. The combination of these technologies has made monitoring easier for both the caregiver and the patient. For instance, mobile applications can show the user’s current health condition and the system can also alert healthcare providers when emergency medical services are needed.

The rise of the 5G mobile network can deliver higher data speeds, lower network latency, and a greater capacity for users. In this case, 5G will benefit both the patient and the telehealth provider by making communication over a distance devoid of delay (Siriwardhana et al., 2020).

Through augmented reality (AR), users can experience the physical world with added digital elements like sounds or visuals. Virtual reality (VR), on the other hand, provides users with 3D views of artificial environments. Although AR/VR in medicine is not widely adopted, it is well-reviewed by several studies (Eckert et al., 2019). Thus, it can serve as an alternative to medical training and treatment in physical settings.

3D printing or additive manufacturing (AM) creates 3D objects based on digital models. During the COVID-19 pandemic, there was an increased demand for protective equipment. AM was used as a supplementary manufacturing process to address emergent supply needs (Tareq et al., 2021). Moreover, digital designs of medical supplies are made available to the public by the National Institutes of Health (NIH) 3D Print Exchange. This online portal by the NIH could promote the use of AM in the medical industry.

We are in the middle of a fourth technological revolution in medicine — Medicine 4.0. These technologies are only some of the key factors driving the transition. Medicine 4.0 will combine current and evolving technologies to create a more efficient healthcare system. Along with this revolution in medicine is the transition into Society 5.0. This society will be one that fully uses digital technology to resolve societal problems and achieve economic development. Simply put, technology won’t only reshape the medical industry, but more importantly, all of society.

## The Status of Telehealth in Southeast Asia

Some SEA countries have turned to telehealth to expand the scope of their healthcare systems. For instance, mobile applications have allowed users to report their health conditions and educate themselves about COVID-19. A real-time information system also helped hospitals in Chiang Mai by integrating their data on one platform, making patient monitoring and hospital referrals easier (Intawong et al., 2021). Thailand has also developed their eHealth Strategy 2017–2026 to employ digital technologies in the health sector effectively. According to the Thailand Ministry of Public Health (2017), central organizations for eHealth management will be established to ensure trust and protection for the health industry and its consumers.

Telehealth has also yielded positive outcomes, especially during the pandemic in Singapore. For instance, medical personnel who have used the mobile messaging platform MyDoc®, strongly agreed that the application should replace current peer-to-peer communication systems in an orthopedic clinic (Daruwalla et al., 2014). Video conferences, mobile apps, and other telehealth platforms have also been found to be practical communication methods for mental health services like counseling and psychoeducation (Zhou et al., 2020). Moreover, the Singapore’s Healthcare Services Act (HCSA) to be implemented in 2022 will replace the Private Hospitals and Medical Clinic Act (PHMCA) of 1980. In this act, the Singapore Ministry of Health (2021) will require a license for telehealth providers to ensure that they comply with safe practice guidelines. This protocol will increase the reliability of telehealth and thereby increase patient confidence in accessing the technology for future consultations.

In the Philippines, increased access to information and communication technologies over the years have led to the rise of telehealth. Because of the pandemic, teleconsultations can now be done through COVID-19 hotlines, websites, and mobile apps launched by several agencies and businesses. These include the Department of Health (DOH), Medgate, KonsultaMD, Medifi, HealthNow, AIDE, DOCPH, Yo-Vivo Health, and Lifeline. Moreover, the House of Representatives filed House Bill No. 7422, or the Congress.gov.ph, 2020, which seeks to establish and develop Philippine telehealth industries using information and communications technologies (ICT) for the delivery of health services. However, this bill is still pending with the Committee on Health. Even before the pandemic, Fernandez-Marcelo et al. (2012) argued that certain factors need to be discussed to develop the telehealth industry. These factors include policies, capability building, and the collaboration of different sectors. Meanwhile, SEA countries like Indonesia, Malaysia, Singapore, Thailand, and Vietnam have already developed guidelines on telehealth (Intan Sabrina and Defi, 2021). The establishment of telehealth in the neighboring countries of the Philippines already serves as proof of the significance of the technology, particularly in this pandemic. Hence, timely implementation and consolidation of telehealth services in the Philippines is of utmost importance.

## The Role of Telehealth in the COVID-19 Pandemic

During the COVID-19 pandemic, healthcare systems were forced to adapt to remote delivery in order to minimize transmission of the virus. The need to accommodate both COVID-19 and COVID-19-free patients has also been a challenge. Many studies suggest that telehealth has improved healthcare provision during the COVID-19 pandemic and recommend it for public safety (Nguyen et al., 2008; Monaghesh and Hajizadeh, 2020; Somsiri et al., 2020; Bagayoko et al., 2014). These studies also discuss how telehealth can contain the pandemic and preserve personal protective equipment. Telehealth has also improved the management of chronic diseases and has proven to save travel time and money (Xu et al., 2018; Somsiri et al., 2020). According to the Philippine Statistics Authority (2020), the health expenditure on senior citizens has reached around 3.43 billion USD in 2020, of which 2.03 billion USD were out-of-pocket expenses while 1.4 billion USD were financed by Philippine Health Insurance Corporation, domestic revenue-based schemes, and other financing sources like those by health maintenance organizations.^1^ By investing in telehealth, nations can better healthcare provision, improve labor productivity and ultimately increase economic performance (Raghupathi and Raghupathi, 2020). The ultimate advantages that telehealth brings to the public are convenience, security, and a safer healthcare alternative during the pandemic.

### Barriers to Telehealth and Some Potential Solutions

Although telehealth offers numerous benefits, some barriers require serious attention for telehealth to succeed. Based on the WHO, lack of policy in SEA is the main barrier to telehealth implementation (Geneva: World Health Organizations, 2011). In the Philippines, for example, there are no existing legal frameworks for the implementation of telehealth (World Health Organizations, 2020). However, different eHealth initiatives have shown promise in breaking this barrier. The DOH has also proposed the Philippine eHealth Strategic Framework and Plan 2014–2020, which includes establishing telehealth infrastructures (Department of Health, 2021). On the legislative side, House Bills 7,422 and 7,153, which support the establishment of ICT and medical bases for eHealth services and solutions, have also been introduced. Furthermore, PhilHealth and its partners will finance applicable eHealth services, as per the National eHealth Steering Committee.

According to the Centers for Disease Control and Prevention (2020), poor internet connectivity, low cellular reception, technological illiteracy, and lack of access to gadgets are barriers to telehealth. Many Philippine schools lack computers, internet access, and trained ICT educators to increase digital literacy according to the Department of Information and Communication Technology, 2014. In Cambodia, only 32.4% of the population with tertiary education uses computers (Nit et al., 2021). Physicians who utilize virtual media in delivering healthcare services need formal training in the use of these media to guarantee outcomes similar to in-person consultations (Nochomovitz and Sharma, 2017). As a solution, telehealth must be included in medical curricula for student-physicians to acquire the necessary education and training. According to Balaji and Clever (2021), it is essential to include telehealth exposure in different medical programs. Moreover, before residency, exposure of medical students to telehealth systems is critical. Proper education on modern ICT tools for older patients is also essential to address the challenges in the use of modern technologies (Holderried et al., 2021).

Since technology is the core foundation of telehealth, the attitude towards accepting the technology is also necessary. Fortunately, a study states that limited evidence shows that older adults, among the major healthcare recipients, are averse to utilizing technology and were found to preferentially use a different form of technology (e.g., television, radio, telephone) compared with young adults (van Houwelingen et al., 2018). To facilitate the adoption of the new technology, providing sufficient support is essential to improve patient confidence in their ability to utilize the technology (Holden and Karsh, 2010). Furthermore, the Technology Acceptance Model (TAM) and its modified version, the Unified Theory of Acceptance and Use of Technology (UTAUT), relates perceived usefulness with increased acceptance and adoption of the new technology (Houwelingen et al., 2018; Schinasi et al., 2021). The TAM and UTAUT were developed to discern how individuals begin to accept and use technological advancements, thereby correlating clinician and patient acceptance of telehealth services.

Internet speeds affect the quality of communication between patients and healthcare providers. The average internet speed in the Philippines is at 2.8 Mbps (20.35USD/Mbps), while Indonesia’s is at 2.2 Mbps (18.83USD/Mbps), and Malaysia’s is at 4.3 Mbps (10.29USD/Mbps) (Salac and Kim, 2016). Several studies report that the quality of home online health consultation systems are interrupted by slow internet speeds that result in poor audio and video quality, connection loss, and patients displeasure (Bernocchi et al., 2016; Dimitropoulos et al., 2017; Eslami Jahromi and Ahmadian, 2018; Almathami et al., 2020). Hence, strengthening internet connectivity and cell reception, educating telecommunication users, and increasing access to gadgets are essential for the implementation of telehealth.

## Discussion

Telehealth is a healthcare revolution. It is not only driven by internet connectivity, but by several other technologies, as well. Though it has been long introduced in the Philippines, its potential hasn’t been fully realized until the pandemic struck. The COVID-19 pandemic has pushed its development in the country further, making it the “new normal” means of delivering healthcare. Unfortunately, telehealth training programs are limited, so distant and secluded communities remain uninformed. Hence, we suggest that local government units participate in the conduct of telehealth training for healthcare providers and information drives for the public. Educational institutions, including medical schools, in the country should also implement courses on both the provision and the use of telehealth into their curricula, to ensure that future healthcare providers and patients have the proper education and training to provide and receive medical care using available ICT tools.

The lack of access to technology necessary to obtain telehealth services also hinders successful telehealth implementation. For patients with limited access to necessary devices, same-day teleconsultations at local clinics may guarantee that all patients receive the medical care they need and deserve. Telehealth services must also be simple and straightforward for patients of all ages to utilize. Finally, we must remember that every community telehealth program is unique, and there is no one-size-fits-all approach to its implementation. Implementation strategies will differ from one healthcare environment to another due to numerous variables.

The establishment of telehealth as an alternative to traditional face-to-face healthcare services can improve the quality of life of many Filipinos. For this reason, the Philippine government must put greater emphasis on its implementation in the country, especially during this time of the pandemic. Collaboration with the government can expedite the implementation process and fill in existing gaps. Overall, it is possible to establish telehealth in the country despite the existing challenges. It is time to improve the country’s healthcare system and give Filipinos equitable access to healthcare. Things might take time, but with proper planning, cooperation, and dedication in keeping everything running, good-quality care may just be beneath our fingertips.

## Footnotes

1
https://psa.gov.ph/pnha-press-release/node/162928
